# Prognostic value of cell division cycle-associated protein-3 in prostate cancer

**DOI:** 10.1097/MD.0000000000034655

**Published:** 2023-09-08

**Authors:** Peng Gu, Minhao Zhang, Xinhua Chen, Jie Du, Lu Chen, Xiaoliang He, Meilin Li

**Affiliations:** a Department of Urology, Xishan People’s Hospital of Wuxi City, Wuxi, Jiangsu, China; b Department of Operating Room, Xishan People’s Hospital of Wuxi City, Wuxi, Jiangsu, China; c Department of Pathology, Xishan People’s Hospital of Wuxi City, Wuxi, Jiangsu, China; d Department of Medicine, Wuxi No. 5 People’s Hospital, Wuxi, Jiangsu, China.

**Keywords:** CDCA3, cell cycle, prognosis, prostate cancer

## Abstract

**Background:**

The study was aimed to explore cell division cycle-associated protein-3 (CDCA3) expression and its correlation with clinicopathological characteristics, and identification of co-expressed genes of CDCA3 in prostate cancer (PCa).

**Methods:**

Data for CDCA3 mRNA expression in PCa were obtained from The Cancer Genome Atlas database. Furtherly, CDCA3 protein expression was examined by immunohistochemistry in 80 cases, including 20 normal prostate samples and 60 PCa samples. Then we used “survival” package to obtain the differentially expressed CDCA3 mRNA associated with prognosis of PCa patients. “pROC” package was used to analyze receiver operating characteristic of CDCA3. We used chi-square test, Kruskal–Wallis test and Wilcoxon rank sum test to identify clinicopathological parameters that correlated with CDCA3 expression. In order to determine the effects of CDCA3 expression and clinicopathological parameters on survival, univariate cox regression analysis was performed. Finally, the co-expressed genes of CDCA3 in PCa were explored by search tool for the retrieval of interacting genes, Kyoto encyclopedia of genes and genomes enrichment analysis and Spearman correlation analysis.

**Results:**

In this study, we found that CDCA3 expression was increased in PCa. PCa patients with higher CDCA3 expression had poor outcomes. In terms of receiver operating characteristic, CDCA3 had an area under the curve of 0.857. High CDCA3 expression was positively correlated with advanced T stage, N stage, Gleason score, and served as an independent predictor of progress free interval in PCa patients. Then 20 proteins closely related to CDCA3 were screened through STRING website. Functional enrichment analysis revealed that, Kyoto encyclopedia of genes and genomes pathway was mainly enriched in cell cycle, including 6 genes, BUB1, CCNA2, CDK1, CDC20, TTK, and CCNB2.

**Conclusion:**

CDCA3 is significantly associated with the prognosis of PCa, which may be an indicator of the diagnosis and prognosis of PCa and a new therapeutic target.

## 1. Introduction

Prostate cancer (PCa) is the second most common solid tumor in men and the fifth cause of cancer mortality,^[1]^ with an estimate of 288,300 new cases and 34,700 deaths in the United States in 2023.^[2]^ Currently, the cornerstones of PCa management include serum prostate-specific antigen (PSA) quantification, digital rectal examination, and systemic transrectal ultrasound-guided biopsies.^[3]^ Molecular biomarkers supplement existing clinicopathologic tools for PCa diagnosis and prognosis by providing additional and valuable information about the biological behavior of PCa. Therefore, the identification of new targets is of great significance to improve the management of PCa patients.

Abnormal regulation of cell cycle will lead to uncontrolled excessive proliferation of cells and promote the formation of cancer, which is of great significance in the occurrence and development of cancer. Therefore, the study of cell cycle regulation-related factors is of great significance for revealing the occurrence and development of cancer.^[4]^ Cell division cycle-associated protein-3 (CDCA3), also known as trigger of mitosis entry 1, is involved in the regulation of cell cycle progression.^[5]^ CDCA3 is a part of the Skp1/Cullin 1/F-box (SCF) ubiquitin ligase (E3) protein complex, which effectively degrades tyrosine kinase wee1 and modulates cell cycle.^[5–7]^ Moreover, CDCA3 emerges with up-regulated expression in a variety of cancers, such as bladder urothelial carcinoma,^[8]^ colorectal cancer,^[9]^ non-small cell lung cancer,^[10]^ hepatocellular carcinoma,^[11]^ and ovarian cancer,^[12]^ and plays a critical role in promoting tumor progression. CDCA3 also has been found to be a good biomarker candidate to predict the prognosis of renal cell carcinoma and kidney renal papillary cell carcinoma patients.^[13,14]^ We have reported that knockdown of CDCA3 potentially suppressed PCa progression via the significant accumulation of p21 and via inhibiting the expression of cyclin D1 by regulating the NF-κB signaling pathway.^[15]^ The study aims to determine whether CDCA3 is associated with prognosis and clinicopathological characteristics in PCa and identify possible downstream target genes co-expressed with CDCA3.

## 2. Materials and methods

### 2.1. Data acquisition

From The Cancer Genome Atlas database (TCGA) data (https://portal.gdc.cancer.gov/), RNA sequencing (RNA-seq) data on PCa and related cancers were obtained. In total, 549 samples were selected as the TCGA cohort including 497 PCa samples and 52 normal samples. Level 3 high-throughput RNA-seq data and corresponding clinicopathological information data were downloaded from the PCa project of the TCGA GDC data portal. RNA-seq data in fragments per kilobase per million format were converted into transcripts per million reads format for comparisons of CDCA3 expression levels between samples.

### 2.2. Survival analysis

The progress free interval (PFI) prognostic value of the CDCA3 mRNA expression level in PCa was analyzed using the “survival” package of R. Based on the median values of CDCA3 expression (transcripts per million reads), patients with PCa were divided into CDCA3-low expression group and CDCA3-high expression group. Results with *P* < .05 were considered statically significant.

### 2.3. Receiver operating characteristic (ROC) curve

CDCA3 expression was analyzed using TCGA database, “ggplot2” package was used to visual CDCA3 expression from TCGA. “pROC” package was used to analyze ROC. The area under curve (AUC)of the ROC curve was generated to evaluate the predictive value of the gene. AUC values closer to 1.0 indicated a better diagnosis, 0.5 to 0.7 indicated a low predictive value, 0.7 to 0.9 indicated moderate predictive accuracy, and >0.9 indicated a high accuracy.

### 2.4. Prostate specimens

The study was performed after approval by the Ethics Committee of Xishan People’s Hospital of Wuxi City (approval no. xs2020ky014). A total of 60 samples were collected to detect CDCA3 expression in PCa. All PCa patients were treated with radical prostatectomy between January 2021 and October 2022. Patients were excluded if they were treated with hormonal therapy, radiation therapy, or chemotherapy prior to surgery, if they lack clinical and follow-up information. In addition, 20 benign prostatic hyperplasia samples were collected to determine CDCA3 expression in normal prostate tissues. Benign prostatic hyperplasia patients were randomly selected from patients treated by transurethral resection of prostate between January 2021 and October 2022.

### 2.5. Immunohistochemistry

Tissues were fixed in 4% paraformaldehyde (room temperature, 12 hours) and embedded in paraffin. Immunohistochemical staining was performed on 4 µm sections of paraffin blocks. After blocking with 3% hydrogen peroxide for 10 minutes at room temperature, the slides were then incubated overnight at 4 °C with primary antibody. The primary antibodies, CDCA3 (diluted 1:150; product no. YT0819; Immunoway, Inc., Plano, TX) was used for staining. The slides were then incubated with the biotinylated goat-anti-rabbit secondary antibody (diluted 1:50; product no. A0208; Beyotime, Inc., Nantong) for 30 minutes at room temperature, and visualized by peroxidase substrate DAB kit (Beyotime, Inc., Nantong) according to the manufacturer’s instructions. The slides were analyzed using an Olympus CKX41 fluorescence microscope (Olympus Corporation, Shinjuku City).

### 2.6. Identification of co-expressed genes

The co-expressed genes of CDCA3 in PCa were analyzed by bioinformatics in TCGA database. Search tool for the retrieval of interacting genes (STRING) is a biological database (https://string-db.org) for constructing protein–protein interaction networks, providing a system-wide view of interactions between each member. Firstly, top 20 proteins closely related to CDCA3 were screened through STRING, and an interaction with a combined score >0.4 was considered statistically significant. In order to investigate functions of the 20 proteins closely related to CDCA3, the gene ontology, and Kyoto encyclopedia of genes and genomes (KEGG) enrichment analysis were performed. We used the “clusterProfiler” package to automate the processes of gene ontology and KEGG term. Finally, spearman correlation analysis was performed on CDCA3 and co-expressed genes by R. We used “ggplot2” package to visual a heat map of CDCA3 with its co-expressed mRNA. Statistical significance was set at *P* < .05.

### 2.7. Statistical analysis

All statistical analyses and the generation of plots were performed using R (4.2.1). The Wilcoxon rank-sum test and Wilcoxon signed-rank test were used to compare the expression of CDCA3 in unpaired samples and paired samples, respectively. The chi-square test, Kruskal–Wallis test and Wilcoxon rank sum test was used to evaluate the correlation between CDCA3 expression and clinicopathological parameters. Univariate analysis was used to analyze the effects of CDCA3 expression and the clinicopathological parameters on survival. *P* < .05 was statistically significant.

Statistical analyses in the part of immunohistochemistry were performed using SPSS 17.0 software. Each value was acquired from at least 3 independent experiments. Normally distributed continuous variables were expressed as mean ± SD, and a 2-tailed unpaired Student *t* test was used to analyze statistical differences between 2 groups. Nonnormally distributed data were expressed in median with interquartile range, and compared by Kruskal–Wallis rank test. The chi-squared test was performed to evaluate categorical variables. *P* < .05 was considered to indicate a statistically significant difference.

## 3. Results

### 3.1. There was a strong correlation between high expression of CDCA3 and bad prognosis in PCa

**Figure 1.**
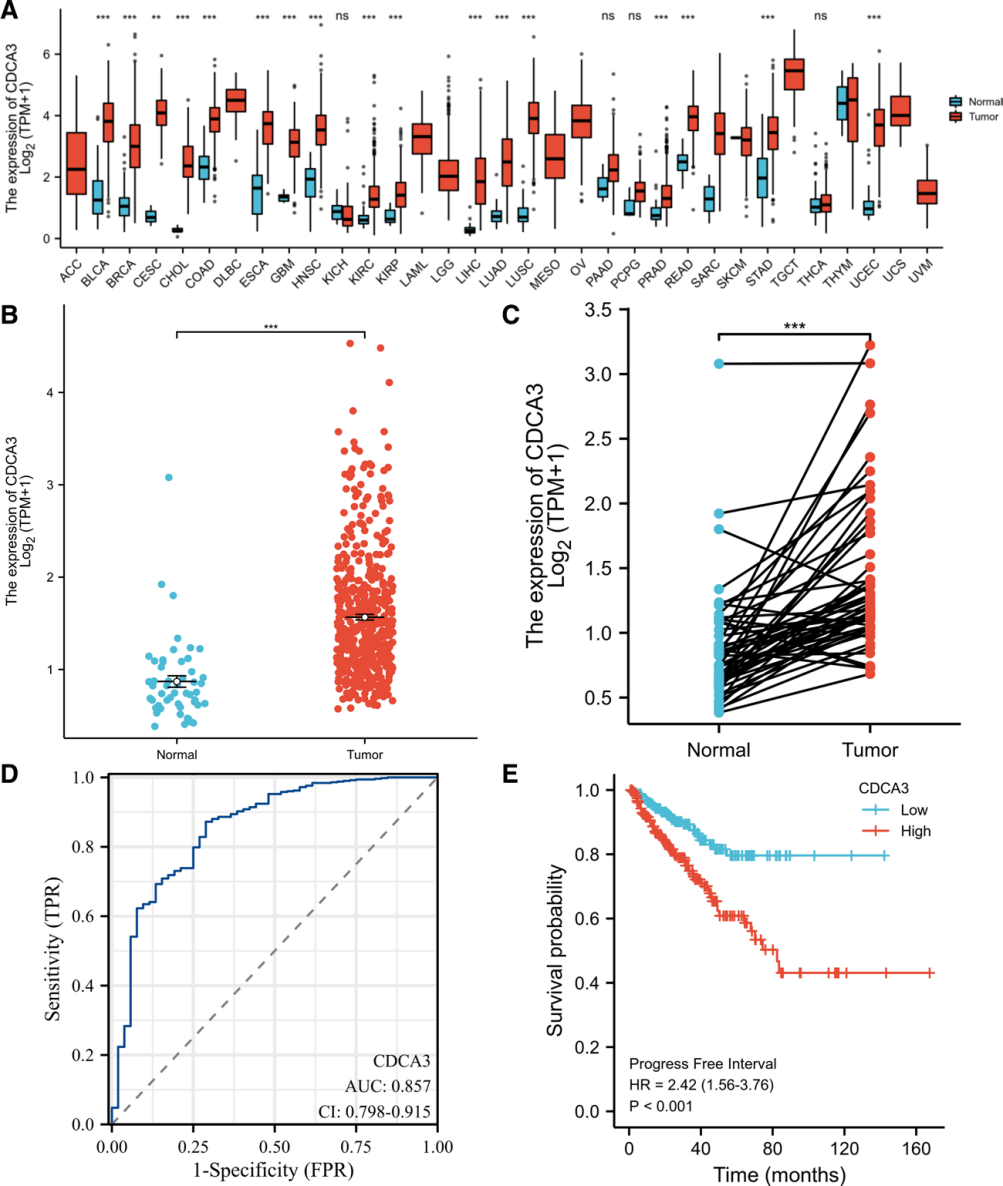
A bad prognosis was linked to upregulation of CDCA3 in prostate cancer (PCa) patients. (A) The expression of CDCA3 in pan-cancer from TCGA database. (B–C) Differential expression analysis of CDCA3 between PCa and normal tissues in TCGA database. (D) Sensitivity and specificity of CDCA3 in the diagnosis of PCa. (E) Progress free interval (PFI) curve of CDCA3 from TCGA database. ns, *P* > .05; **P < .01; ***P < .001. TCGA = The Cancer Genome Atlas.

Investigating the differential expression of CDCA3 between human tumors and normal tissues, the expression levels of CDCA3 in pan-cancer were analyzed using the TCGA database. The results showed that CDCA3 was highly expressed in prostate adenocarcinoma, and other cancer types compared with each normal tissue (Fig. 1A). Visual analysis showed that the expression of CDCA3 in PCa was significantly higher than that in normal tissues (Fig. 1B). The upregulation of CDCA3 expression was also observed in PCa tissues compared to that in paired normal samples (Fig. 1C). ROC curve was used to analyze the sensitivity and specificity of CDCA3 in the diagnosis of PCa. The sensitivity, specificity, AUC, and cutoff value were 0.872, 0.712, 0.857, and 0.913, respectively (Fig. 1D). We found that the CDCA3 expression status could serve as a potential predictor for PCa in TCGA database. The Kaplan–Meier curve was generated to evaluate the prognostic value of CDCA3 with respect to the PFI in CDCA3 expression subgroups in PCa patients. High CDCA3 expression in PCa patients associated with a worse PFI (HR = 2.42, *P* < .001, Fig. 1E).

### 3.2. Clinicopathological factors associated with CDCA3 in PCa

**Figure 2.**
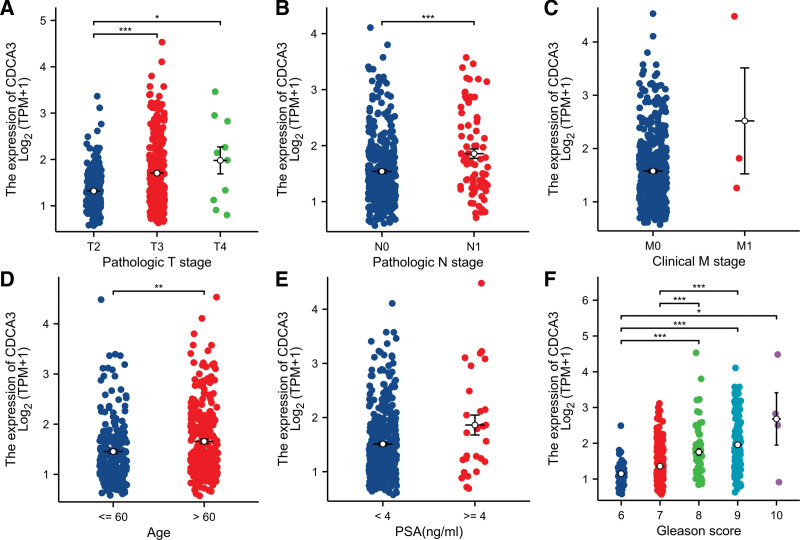
Clinicopathological factors associated with CDCA3 in prostate cancer (PCa) patients in TCGA database. (A–F) CDCA3 mRNA expression with clinicopathological parameters of patients with PCa, including pathologic T stage, pathologic N stage, clinical M stage, age, PSA and Gleason score. *P < .05; **P < .01; ***P < .001. CDCA3 = cell division cycle-associated protein-3, PSA = prostate-specific antigen.

When analyzing the correlation between CDCA3 expression and clinicopathological characteristics of PCa patients. 497 prostate samples were downloaded from the TCGA database, including the low CDCA3 expression group (n = 248) and the high CDCA3 expression group (n = 249). Chi-squared test showed that the high CDCA3 expression was positively correlated with pathologic T3–T4, pathologic N1 and Gleason score 8–10 (*P* < .05, Table 1). As shown in Figure 2, consistent results were obtained that the high CDCA3 expression was positively correlated with advanced pathologic T stage, pathologic N stage, Gleason score and age using the Kruskal–Wallis test and Wilcoxon rank sum test.

**Table 1:** Clinicopathological features of prostate cancer patients associated with CDCA3 expression.

Characteristic	Low expression of CDCA3	High expression of CDCA3	*P*
n	248	249	
Pathologic T stage, n (%)			<.001
T2	118 (24.1%)	69 (14.1%)	
T3–T4	126 (25.7%)	177 (36.1%)	
Pathologic N stage, n (%)			.039
N0	171 (40.3%)	174 (41%)	
N1	29 (6.8%)	50 (11.8%)	
Clinical M stage, n (%)			1
M0	221 (48.3%)	234 (51.1%)	
M1	1 (0.2%)	2 (0.4%)	
Age, n (%)			.164
≤60	119 (23.9%)	104 (20.9%)	
>60	129 (26%)	145 (29.2%)	
PSA (ng/mL), n (%)			0.443
<4	215 (48.9%)	198 (45%)	
≥4	12 (2.7%)	15 (3.4%)	
Gleason score, n (%)			<.001
6–7	185 (37.2%)	107 (21.5%)	
8–10	63 (12.7%)	142 (28.6%)	

*P <*.05, and the results were statistically significant.

CDCA3 = cell division cycle-associated protein-3, PSA = prostate-specific antigen.

To examine the effects of CDCA3 expression and clinicopathological parameters on survival, we used univariate cox regression analyses. The result revealed that the pathologic T stage, pathologic N stage, PSA, Gleason score, and CDCA3 expression impacted PFI of PCa patients independently, as shown in Table 2.

**Table 2:** A univariate analysis of clinicopathological parameters in patients with prostate cancer on progression free interval (PFI).

Characteristics	Total(N)	Univariate analysis	
		Hazard ratio (95% CI)	*P*-value
Pathologic T stage	490		
T2	187	Reference	
T3–T4	303	3.676 (2.078–6.505)	<.001
Pathologic N stage	424		
N0	345	Reference	
N1	79	1.812 (1.110–2.956)	.017
Clinical M stage	458		
M0	455	Reference	
M1	3	3.670 (0.508–26.512)	.198
Age	497		
≤60	223	Reference	
>60	274	1.276 (0.845–1.927)	.246
PSA (ng/mL)	440		
<4	413	Reference	
≥4	27	4.274 (2.132–8.566)	<.001
Gleason score	497		
6–7	292	Reference	
8–10	205	4.594 (2.903–7.269)	<.001
CDCA3	497		
Low	248	Reference	
High	249	2.237 (1.447–3.458)	<.001

*P* < .05, and the results were statistically significant.

CDCA3 = cell division cycle-associated protein-3, PSA = prostate-specific antigen.

### 3.3. CDCA3 expression and clinicopathological features in patients with PCa by immunohistochemistry

**Figure 3.**
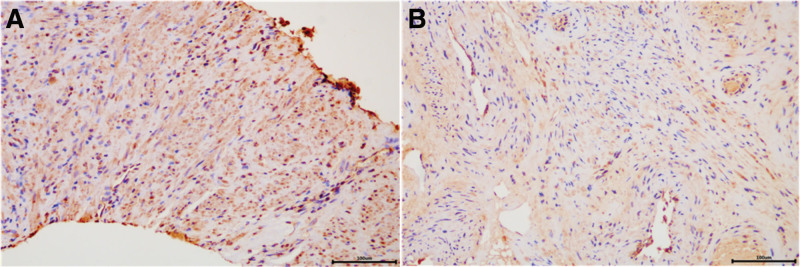
Immunohistochemical staining of CDCA3 in human prostate tissues. (A) Prostate cancer samples. (B) Normal prostate samples. Scale bar indicates 100 μm. CDCA3 = cell division cycle-associated protein-3.

To evaluate CDCA3 protein expression in PCa, 20 normal prostate samples and 60 PCa samples were analyzed by immunohistochemistry. CDCA3 high expression was found in 15.0% (3/20) of normal prostate samples. In addition, CDCA3 high expression was detected in 66.7% (40/60) of PCa samples (Fig. 3). It was also found that CDCA3 high expression was significantly associated with PCa by chi-square test (*P* < .001). Furthermore, CDCA3 high expression was significantly positive related to PSA, tumor stage and Gleason score (Table 3).

**Table 3:** Correlation between CDCA3 expression and clinicopathological factors by immunohistochemistry.

Characteristic	Low expression of CDCA3	High expression of CDCA3	*P*
n	20	40	
Age (years, x±s)	73.95 ± 5.22	74.38 ± 5.74	.780
PSA (ng/mL，M [IQR])	9.69 (9.03)	22.47 (43.47)	<.001
T stage			0.026
T2	18	25	
T3–T4	2	15	
Gleason score			0.017
6–7	15	17	
>7	5	23	

*P* <.05, and the results were statistically significant.

CDCA3 = cell division cycle-associated protein-3, IQR = interquartile range.

### 3.4. Identification of co-expressed genes

**Figure 4.**
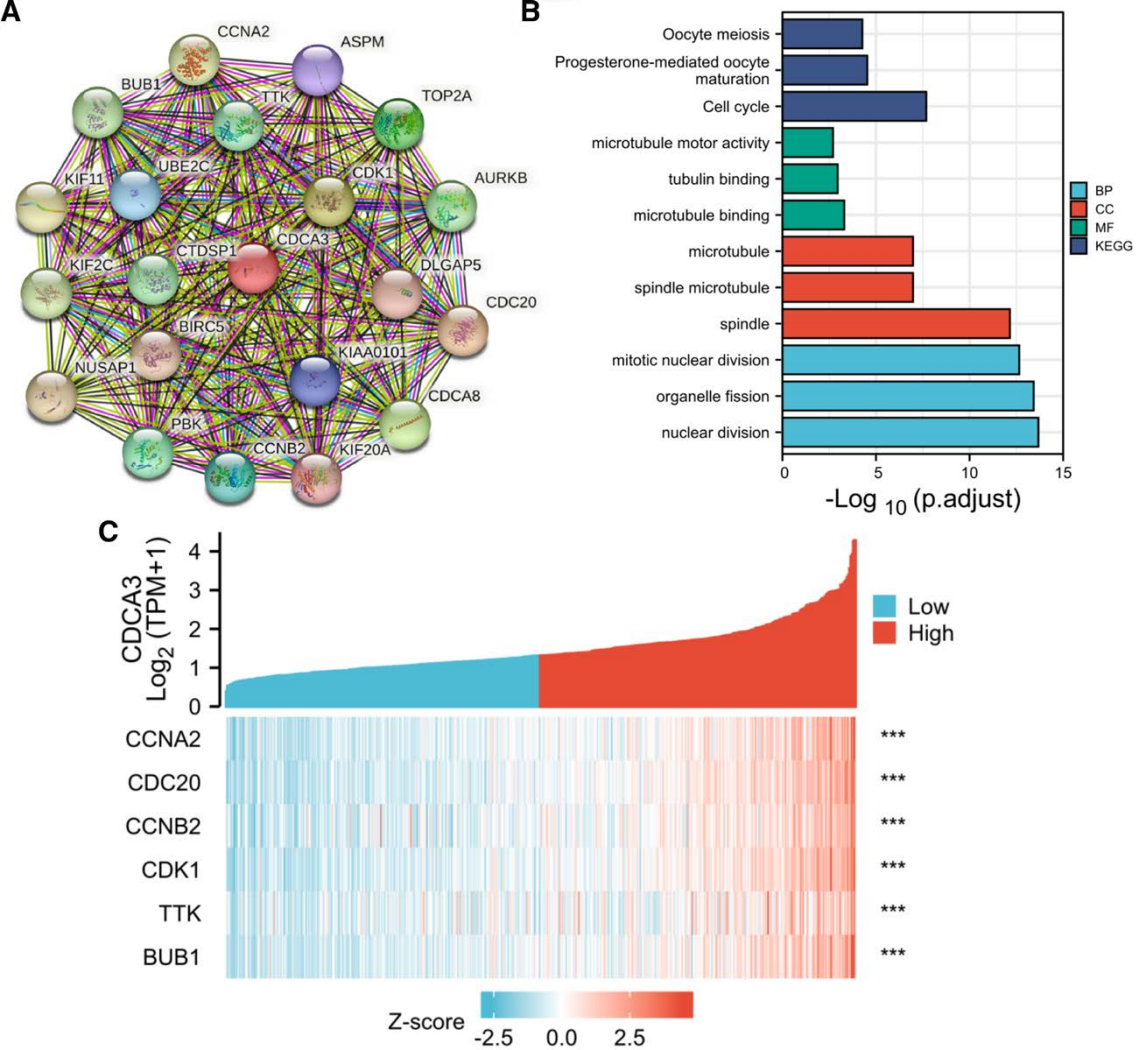
Identification of co-expressed genes of CDCA3 in prostate cancer. (A) The protein–protein interaction (PPI) networks of CDCA3 and top 20 closely related proteins. (B) Gene ontology (GO) and Kyoto encyclopedia of genes and genomes (KEGG) enrichment analysis. (C) A heat map of CDCA3 and its co-expressed mRNA. ^***^P < .001. CDCA3 = cell division cycle-associated protein-3.

First of all, top 20 proteins (CCNA2, BUB1, TTK, ASPM, TOP2A, KIF11, UBE2C, CDK1, AURKB, KIF2C, CTDSP1, DLGAP5, CDC20, BIRC5, NUSAP1, KIAA0101, CDCA8, KIF20A, CCNB2, PBK) closely related to CDCA3 were screened through STRING website. The protein–protein interaction networks was shown in Figure 4A. KEGG pathway analysis indicated that the 20 genes were mainly enriched in cell cycle (Fig. 4B). There were 6 genes enriched in the cell cycle, including BUB1, CCNA2, CDK1, CDC20, TTK, and CCNB2(Table 4). Spearman correlation analysis results indicated significant and positive correlations between CDCA3 and the 6 genes (Table 5), and we called them co-expressed genes. The heat map of CDCA3 and its co-expressed mRNA was shown in Figure 4C.

**Table 4:** GO and KEGG pathway analysis of the top 20 genes closely related to CDCA3.

ID	Description	*P*-value	Gene ID	Count
hsa04110	Cell cycle	9.39e−10	BUB1/CCNA2/CDK1/CDC20/TTK/CCNB2	6
hsa04914	Progesterone-mediated oocyte maturation	2.66e−06	BUB1/CCNA2/CDK1/CCNB2	4
hsa04114	Oocyte meiosis	7.36e−06	BUB1/CDK1/CDC20/CCNB2	4

*P* < .05, and the results were statistically significant.

CDCA3 = cell division cycle-associated protein-3, GO = gene ontology, KEGG = Kyoto encyclopedia of genes and genomes.

**Table 5:** Spearman correlation analysis between CDCA3 and co-expressed genes in TCGA database.

Target gene	Co-expressed gene	Spearman coefficient	*P*
CDCA3	CCNA2	0.788	<.001
CDCA3	CDC20	0.837	<.001
CDCA3	CCNB2	0.680	<.001
CDCA3	CDK1	0.809	<.001
CDCA3	TTK	0.562	<.001
CDCA3	BUB1	0.753	<.001

*P* < .05, and the results were statistically significant.

## 4. Discussion

Recent studies of high throughput gene chips for analysis of patient average and tumor tissue samples have provided us with an opportunity to discover and explore the entire molecular landscape of tumors at various levels, from copy number changes and somatic mutations at the genomic level to altered gene expression at the transcriptional level.^[16]^ In our previous study, we have analyzed microarray data of localized PCa, metastatic PCa and normal prostate tissue samples from clinical specimens to discover key molecular changes in PCa development and metastasis.^[17]^ We also have reported CDCA family (CDCA1–8) had important clinical relevance in the development and progression of prostate cancer by bioinformatics analysis.^[18]^ It has reported that the expression of CDCA1 were more than 3-fold up-regulated in human PCa tissue compared to the adjacent normal tissues.^[19]^ For PCa, molecular targeted therapy and immunotherapy have played certain therapeutic roles in recent years. Additionally, CDCA1-derived peptide vaccine treatment was tolerable and might effectively induce peptide specific cytotoxic T lymphocytes for castration resistant prostate cancer patients in a phase I clinical trial.^[20]^ CDCA2, another member of the CDCA family, was overexpressed in primary PCa and positively correlated with poor prognosis in patients with PCa.^[21]^ Similarly, CDCA5 expression affected the prognosis of PCa patients, it has revealed that patients with high CDCA5 expression had a high initial PSA. Additionally, seminal vesicle invasion and lymph node metastasis were more likely to occur.^[22]^ CDCA6, also known as CBX2, has reported up-regulated in metastatic castration resistant prostate cancer and elevated expression was correlated with poor clinical outcome in PCa cohorts.^[23]^ In a study of CDCA8, high CDCA8 expression predicted poor prognosis in PCa patients, and CDCA8 expression was higher in high-grade PCa.^[16]^ Although, we have reported that CDCA3 knockdown in DU145 and PC-3 cells led to decreased cell proliferation and increased apoptosis,^[15]^ the prognostic value of CDCA3 in patients and its relationship with clinicopathological characteristics in PCa remain unclear.

In our study, according to the TCGA database and results of immunohistochemistry, as compared to prostate normal samples, PCa had higher level of CDCA3 expression. Upregulated CDCA3 expression in PCa was related to advanced clinicopathological parameters (T stage, N stage, and Gleason score in TCGA database; PSA, T stage and Gleason score in immunohistochemistry) and bad PFI prognosis, and CDCA3 had a high diagnostic rate. Furthermore, the CDCA3 was an independent factor of PFI prognosis according to univariate regression analysis, suggesting that patients with PCa might benefit from the use of CDCA3 as a biomarker for diagnosis and prognosis. Based on STRING and KEGG pathway enrichment analysis, it showed CDCA3 and the 6 genes closely related to CDCA3 mainly enriched in cell cycle signaling pathway. Spearman correlation analysis indicated significant and positive correlations between CDCA3 and the 6 genes, and we called them co-expressed genes (BUB1, CCNA2, CDK1, CDC20, TTK, and CCNB2).

CDCA3 is a cytosolic protein required for proper activation of CDK1/cyclin B and mitotic entry. Degradation of CDCA3 during G1 allows for wee1 accumulation during interphase, thereby providing a critical link between the anaphase promoting complex and SCF pathways in regulation of CDK1/cyclin B activity and thus mitotic entry and exit.^[5]^ It was reported that everolimus resistance in prostate cancer cells was characterized by an increased level of cdk1 and cyclin B, driving the cells towards G2/M. Reduction of cdk1 or cyclin B considerably diminished tumor growth activity.^[24]^ BUB1, a mitotic serine/threonine kinase, has multiple functions in chromosome segregation. It has been found that BUB1 phosphorylates Cdc20 and histone H2A, resulting in active transcription in human cells. BUB1 was also found to be deregulated and linked to tumorigenesis.^[25]^ In PCa, BUB1 was significantly upregulated. Kaplan–Meier survival curves demonstrated that a high level of BUB1 was significantly associated with poor prognostic. Altogether, BUB1 is a potential tumor-promoting gene and partly renders prostate cancer cells the docetaxel resistance via circ_0004087/SND1/MYB/BUB1 axis.^[26]^ CCNA2 is a type of cycling protein, which was first detected in primary liver cancer cells, which appeared in the late G1 and could bind to CDK2 and CDC2.^[27]^ In PCa, CCNA2 was significantly associated with biochemical recurrence, disease-free survival and overall survival rate of PCa patients. The ability of cancer cells proliferation, invasion and metastasis was decreased, and cells were arrested in the S phase after down-regulated expression of CCNA2 in PCa cell lines.^[28]^ CDC20, a key E3 ligase, is a regulator of cell cycle checkpoint that activates the anaphase promoting complex. Previous research has identified CDC20 was over expressed in PCa and correlated with postoperative Gleason scores. Over expression of CDC20 may be a potential biomarker for prediction of biochemical recurrence after radical prostatectomy of clinically localized PCa.^[29]^ The present study revealed that CDC20 and PTTG1 contribute more to migration, progression, and poorer prognoses in metastatic prostate cancer (mPCa) compared with PCa. CDC20 and PTTG1 could represent therapeutic targets in mPCa medical research and clinical studies.^[30]^ The protein kinase TTK is a dual specificity kinase that can phosphorylate not only tyrosine but also serine/threonine residues. The functions of TTK include promoting mitotic checkpoint complex formation, regulating cytokinesis, responding to DNA damage, and facilitating proper chromosome alignment.^[31]^ It was reported that expression of the TTK protein kinase was significantly higher in mPCa cells lines and advanced PCa tissues with high Gleason scores. TTK may inhibit the proliferation of prostate cancer and the progression of prostate cancer by inhibiting the expression of CDK2 and CCNE1 complex.^[32]^

## 5. Conclusion

To conclude, our study combined bioinformatics analysis and immunohistochemistry suggested that CDCA3 was over expressed in PCa. It was also significantly associated with the prognosis of prostate cancer, which may be an indicator of the prognosis of PCa and a new therapeutic target. Our next step will be to explore the relationship between CDCA3 and its co-expressed genes, BUB1, CCNA2, CDK1, CDC20, TTK, and CCNB2. Furthermore, it is determined which co-expressed gene could act as the downstream target of CDCA3.

## Author contributions

**Conceptualization:** Minhao Zhang, Lu Chen, Xiaoliang He, Meilin Li.

**Data curation:** Peng Gu, Minhao Zhang, Xinhua Chen, Jie Du, Lu Chen.

**Formal analysis:** Peng Gu, Minhao Zhang.

**Funding acquisition:** Peng Gu, Minhao Zhang, Xiaoliang He.

**Investigation:** Peng Gu, Minhao Zhang, Lu Chen, Meilin Li.

**Methodology:** Peng Gu, Minhao Zhang, Xinhua Chen, Jie Du, Lu Chen, Meilin Li.

**Project administration:** Peng Gu, Minhao Zhang, Xinhua Chen, Jie Du, Meilin Li.

**Resources:** Peng Gu, Minhao Zhang, Lu Chen.

**Software:** Peng Gu, Minhao Zhang, Xinhua Chen, Lu Chen.

**Supervision:** Peng Gu, Minhao Zhang, Jie Du, Xiaoliang He, Meilin Li.

**Validation:** Peng Gu, Minhao Zhang.

**Visualization:** Peng Gu, Minhao Zhang.

**Writing – original draft:** Peng Gu, Minhao Zhang.

**Writing – review & editing:** Peng Gu, Minhao Zhang, Xiaoliang He, Meilin Li.
